# Textilome abdominal, à propos d'un cas

**DOI:** 10.11604/pamj.2015.21.244.7194

**Published:** 2015-08-05

**Authors:** Driss Erguibi, Robleh Hassan, Mohamed Ajbal, Bouchaib Kadiri

**Affiliations:** 1 Chirurgie Viscérale, Service Aile I, Centre Hospitalier Universitaire IBN Rochd, Universite Hassan II, Casablanca, Maroc

**Keywords:** Textilome, chirurgie abdominale, compresses, Textiloma, abdominal surgery, gauze pads

## Abstract

Le textilome, également appelé gossybipomas, est une complication postopératoire très rare. Il peut s'agir d'un corps étranger composé de compresse(s) ou champ(s) chirurgicaux oubliés au niveau d'un foyer opératoire. Ils sont plus souvent asymptomatiques, et difficile à diagnostiquer. En particulier, les cas chroniques ne présentent pas de signes cliniques et radiologiques spécifiques pour le diagnostic différentiel. L'anamnèse est donc indispensable pour le diagnostic vu que les signes cliniques ne sont pas concluants. Le cliché d'abdomen sans préparation est peu contributif, l’échographie est fiable. La tomodensitométrie permet un diagnostic topographique précis, mais ce n'est pas toujours le cas. Certaines équipes proposent des explorations par IRM. Nous rapportons un cas de textilome intra abdominal, chez une patiente de 31 ans opérée il y a 8 ans pour grossesse extra-utérine, chez qui la TDM abdomino-pelvienne a évoqué un kyste hydatique péritonéale sans localisation du foie. Traitée par extrait d'un petit champ de 25x15cm et adhérant au sigmoïde. Le but de ce travail est de mettre en évidence le problème de diagnostic de cette pathologie et l'importance de la laparotomie exploratrice.

## Introduction

Le textilome, également appelé gossybipoma, est une complication postopératoire très rare mais bien connue. Gossybipoma est un terme dérivé de gossypium signifiant coton en Latin et boma signifiant lieu de cachette en Swahili. Il est utilisé pour décrire un corps étranger composé de compresse(s) ou champ(s) chirurgicaux oubliée au niveau d'un foyer opératoire [1, 2]. C'est une complication peu fréquente de la chirurgie abdominale et pelvienne, difficile à estimer [2].

## Patient et observation

**Figure 1:**
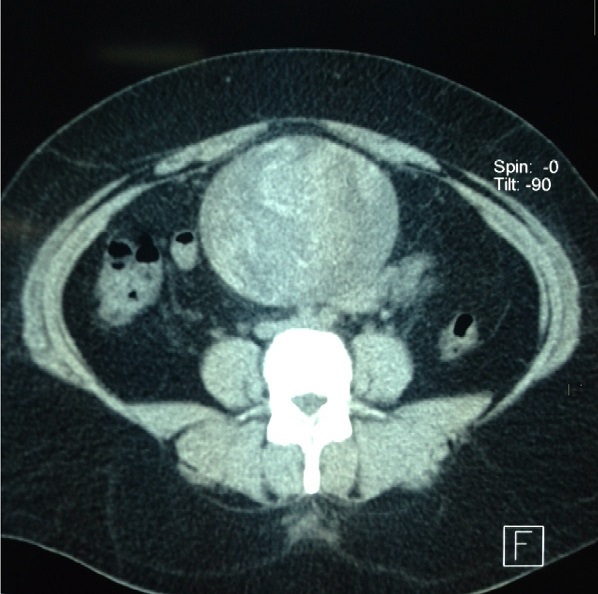
Coupe transverse d'une TDM A-P évoquant un kyste hydatique péritonéal type IV

**Figure 2:**
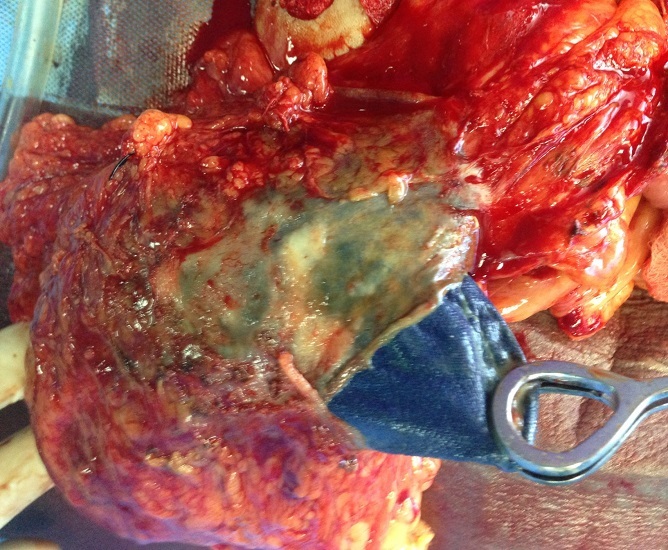
Le champ opératoire extrait de l'abdomen

Patiente âgée de 31 ans, opérée auparavant d'une grossesse extra-utérine par voie médiane sous ombilicale il y a 8 ans, est hospitalisée pour laparotomie exploratrice d'une masse abdomino-pelvienne associée à des douleurs abdominale sans troubles de transit ni troubles urinaires. L'examen clinique montre une patiente en bon état général, apyrétique à 37^°^5 avec une palpation d'une masse para ombilicale mobile par rapport au plan superficiel et profond. La radiographie du thorax était normale. L’échographie abdominale a montré une énorme formation péritonéale hétérogène atypique calcifiée par endroits (Tératome atypique). La tomodensitométrie abdominale a évoqué un kyste hydatique péritonéal type IV sans localisation hépatique (Figure 1). La patiente a bénéficié d'une extraction d'un petit champ opératoire (Figure 2) au colon sigmoïde et enveloppe par le grand épiploon. Les suites post opératoire étaient simples.

## Discussion

Le textilome est une lésion formée suite à l'oubli d'un corps étranger textile lors d'une intervention chirurgicale. Le terme plus académique de gossybipoma témoigne de la réaction inflammatoire induite par un corps textile au contact des tissus qui aboutit à la constitution d'un granulome inflammatoire [3]. La fréquence rapportée dans la littérature est de 1/1 000 à 1/10 000 [4]. La revue de la littérature (117 des cas publiés de 1952 à 1993) insiste sur la prédominance des Textilomes intrapéritonéaux (52%), mais d'autres sites sont concernés: gynécologiques (22%), urologiques et vasculaires (10%), osseux et rachidiens (6%), divers (10%). L'oubli de matériel reste la hantise du chirurgien lors de toute intervention et l’évolution pour le patient peut être dramatique. En effet, dans la revue de littérature de Le Neel et al [5], l'exérèse du textilome aboutit certes à la guérison sans complication chez 70 patients (59,8%), mais les complications ont aggravé l’évolution de 25 malades (21,3%), et 22 patients sont décédés (18,9%). Vingt et un des 22 décès sont à imputer aux Textilomes abdominaux et concernent des Textilomes symptomatiques reconnus tardivement, ayant nécessité des gestes plus agressifs (résection intestinale et/ou colique) avec un pourcentage non négligeable de complications sévères, en particulier septique. Sur le plan physiopathologique, les fibres de textile provoquent dès la 24^e^ heure une réaction inflammatoire avec exsudation suivi par la formation d'un tissu de granulation (8^e^ jour), enfin la fibrose s'organise à partir du 13^e^ jour. Cette évolution explique, en absence d'infection, les possibilités d'enkystement voire des calcifications avec une tolérance parfois longue [3]. La découverte du textilome abdominale est généralement tardive [6]. L'anamnèse est donc essentielle dans l’élaboration du diagnostic. La clinique manque de spécificité. Elle associe des troubles chroniques du transit à des syndromes sub-occlusifs à répétition, comme dans notre observation.

Ces troubles pourraient être liés à des phénomènes de digestion du corps étranger ou à une désinvagination spontanée. Radiologiquement, le cliché d'abdomen sans préparation est peu contributif, comme souvent dans les syndromes pré-occlusifs. L’échographie est fiable et elle montre de multiples bulles d'air extra-digestives ou intra- lésionnelles sans notion d'infection. Ces bulles correspondent à l'air enchâssé dans les mailles d'une compresse en coton ou d'un champ. Les calcifications sont souvent inexistantes [7]. La tomodensitométrie permet un diagnostic topographique préopératoire mais peut évoquer d'autre diagnostic, comme dans notre observation (Figure 2). Elle réalise en même temps une exploration complète de la cavité abdominale à la recherche de complications (fistules, pneumopéritoine, abcès). Certaines équipes proposent des explorations par IRM [6, 8]. En effet le textilome abdominale peut mimer une tumeur conjonctive et l'intestin grêle est une localisation fréquente des formes primitives de lymphome. Le textilome peut être confondu avec un adénocarcinome colique [9]. Le contexte clinique et l'altération de l’état général présentés en cas de tumeur chez un patient jeune aident à redresser le diagnostic, ce qui n'a pas existé dans notre cas. D'autre part, la corrélation entre les explorations échographique, tomodensitométrique et macroscopique permettent de mieux comprendre et analyser les images. Le comptage des compresses et des champs par le chirurgien en début et fin d'intervention reste un moyen efficace mais encore insuffisant. Aux Etats unis, l'utilisation de compresses marquées radio-opaques dès 1940 selon les recommandations de Cr Ossen, a contribué de façon significative à limiter ce type d'incident [5].

## Conclusion

Textilome est une lesion grave et non neglegeable dans la chirurgie abdominale et gynecologique. Le comptage des compresses et des champs par le chirurgien en debut et fin d'intervention est le seul moyen de diminue.
